# Transurethral versus suprapubic catheterization to test urethral function in rats

**DOI:** 10.1038/s41598-021-93772-x

**Published:** 2021-07-13

**Authors:** Kristine Janssen, Kangli Deng, Steve J. A. Majerus, Dan Li Lin, Brett Hanzlicek, Robert S. Butler, Carl H. van der Vaart, Margot S. Damaser

**Affiliations:** 1 Advanced Platform Technology Center, Louis Stokes Cleveland VA Medical Center, Cleveland, OH USA; 2 Biomedical Engineering Department, Lerner Research Institute, Cleveland Clinic, 9500 Euclid Avenue ND 20, Cleveland, OH 44195 USA; 3 Division Woman and Baby, University Medical Center Utrecht, Utrecht, The Netherlands; 4 Department of Electrical Engineering and Computer Science, Case Western Reserve University, Cleveland, OH USA; 5 Glickman Urological and Kidney Institute, Cleveland Clinic, Cleveland, OH USA; 6 Department of Quantitative Health Sciences, Lerner Research Institute, Cleveland Clinic, Cleveland, OH USA

**Keywords:** Biological techniques, Urology

## Abstract

Transurethral and suprapubic catheterization have both been used to test urethral function in rats; however, it is unknown whether these methods affect urethral function or if the order of catheterization affects the results. The aim of this cross-over designed experiment was to compare the effects of catheterization methods and order on leak point pressure (LPP) testing. LPP and simultaneous external urethral sphincter electromyography (EUS EMG) were recorded in anesthetized female virgin Sprague-Dawley rats in a cross-over design to test the effects of transurethral and suprapubic catheterization. There was no significant difference in peak bladder pressure during LPP testing whether measured with a transurethral or suprapubic catheter. There was no significant difference in peak bladder pressure between the first and second catheter insertions. However, peak EMG firing rate, as well as peak EMG amplitude and EMG amplitude difference between peak and baseline were significantly higher after the first catheter insertion compared to the second insertion, regardless of the catheter method. Our results suggest that route of catheterization does not alter urethral function, e.g. create a functional partial outlet obstruction. Either catheterization method could be used for LPP and/or EUS EMG testing in rats.

## Introduction

Stress urinary incontinence (SUI) is prevalent among women, often requiring surgical intervention^1^. Despite this high prevalence, only a few treatment options, including pessaries, Kegel exercises, injection of bulking agents, and surgery, are available^2,3^. Pharmacological treatments are limited and none are approved in the United States. The mainstay of surgery, a mid-urethral sling using polypropylene mesh, is effective but can result in numerous complications^4^. With warnings from the U.S. Food and Drug Administration and withdrawal of products from the market, new treatment options are needed. Development and optimization of animal models of SUI for preclinical testing of novel therapies are essential to improving treatment options for women.

A number of animal models of SUI have been developed in recent years, the most common of which is in female rats^5^. Several different outcomes are utilized to assess SUI in animals, including leak point pressure (LPP) and external urethral sphincter (EUS) electromyography (EMG)^6^. LPP is the bladder pressure at which urethral pressure is exceeded, resulting in leakage of urine and thus, is a measure of the closure ability of the urethra. EUS EMG assesses urethral neuromuscular function during voiding and/or during LPP measurements^7^. These two outcomes together best represent the most direct method of determining urethral function in animal models.

Placement of a bladder catheter is necessary to measure bladder pressure during filling, voiding and LPP testing. Both suprapubic and transurethral catheters have been utilized in rats for LPP and EUS EMG testing^8–10^; however, no head-to-head comparison has been done. Clinically, larger caliber transurethral cystometry catheters can increase LPP and prevent leakage of urine during urodynamic testing^11^, likely due to a partial urethral obstruction and/or irritation or disturbance of the integrity of the mucosal seal^12^. Suprapubic bladder catheterization may prevent this but requires a more invasive procedure with attendant complication risks. On the other hand, in an animal model, placement of a transurethral catheter could contribute to consistency of EUS EMG recordings. Since most of rat continence is sourced in urethral somatic muscles, cerebral control, urethral smooth muscles and mucosal seal coaptation, stabilizing the urethra, preventing urethral hypermobility, with electrodes and a catheter is not likely to impact urethral function^12^. This is in contrast to female SUI, in which urethral hypermobility is the most likely pathophysiological mechanisms for SUI.

The aim of this cross-over designed study was to compare transurethral versus suprapubic catheterization during urethral function testing in female rats and to test whether catheterization method affects LPP or simultaneous EUS EMG results.

## Results

**Figure 1:**
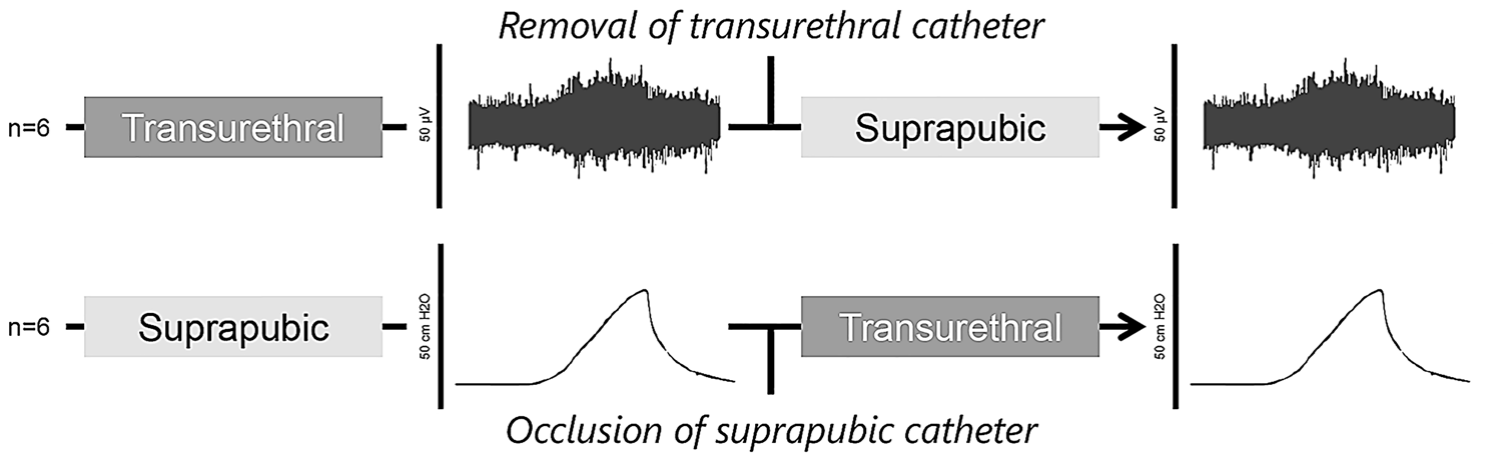
Experimental design. Rats underwent initial catheterization, followed by leak point pressure (LPP) with simultaneous external urethral sphincter (EUS) electromyography (EMG) testing. After initial transurethral catheterization, the catheter was removed and a suprapubic catheter was placed. After initial suprapubic catheterization, the catheter was shortened and occluded and a transurethral catheter was placed. This was followed by repeated LPP with simultaneous EUS EMG testing.

**Figure 2:**
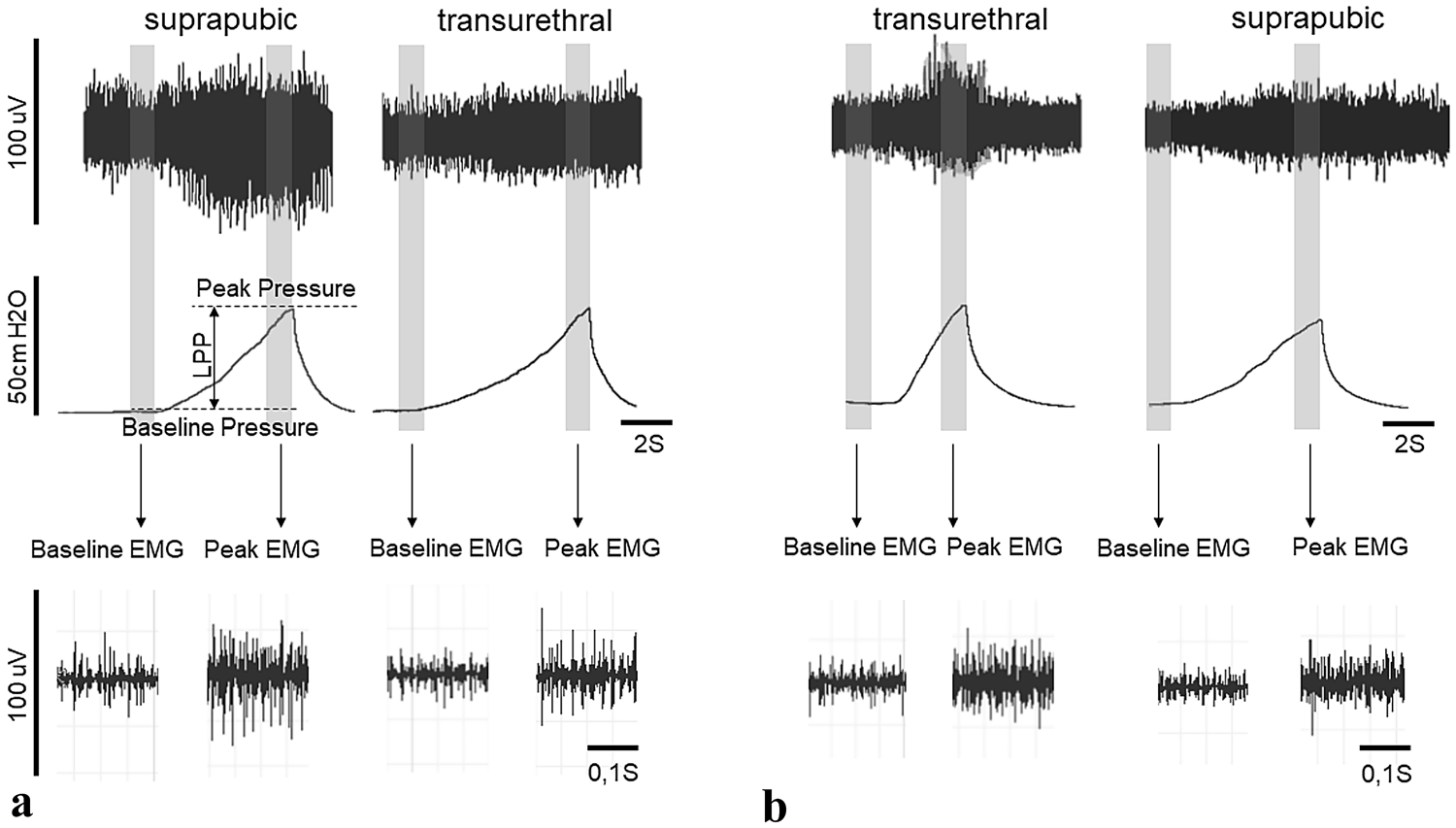
Representative examples of LPP and EMG recording. Both examples represent measurements recorded using PowerLab 8/35 system (LabChart v. 7 software) from one animal each, using either a suprapubic catheter first and a transurethral catheter second (**a**), or a transurethral catheter first and a suprapubic catheter second (**b**). One second segments (shaded region) were used to calculate bladder pressure, external urethral sphincter electromyography firing rate and amplitude at baseline and peak. Leak point pressure (LPP) was calculated by subtracting baseline pressure from peak pressure.

Urethral function was tested with both catheter methods in all twelve otherwise unmanipulated age-matched female Sprague-Dawley rats using a crossover design (Fig. 1). When bladder pressure was increased by pressing down on top of the bladder during LPP testing, EUS EMG firing rate was visibly increased, indicating the presence of a guarding reflex (Fig. 2a, b).

### Leak point pressure (LPP)

**Figure 3:**
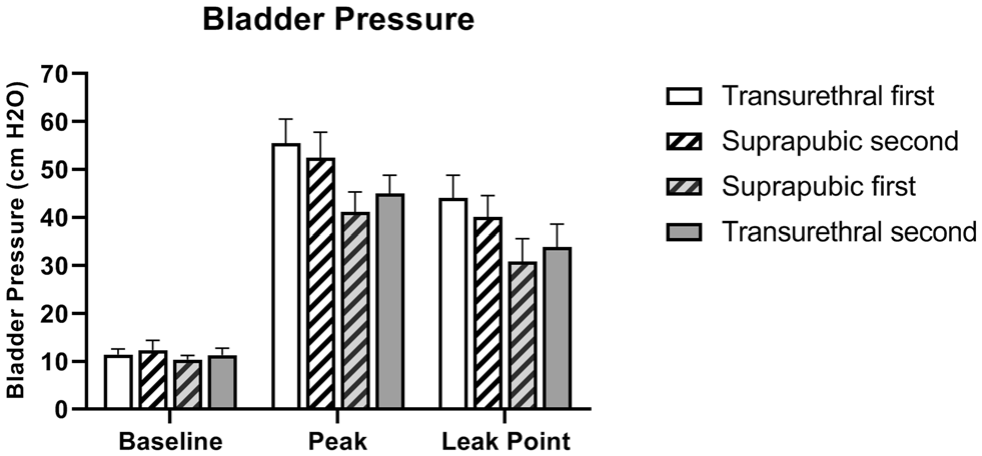
Bladder pressure during LPP testing. Baseline pressure, peak bladder pressure, and Leak Point Pressure grouped by order of catheterization. Repeated measures ANOVA did not reveal any significant interactions between catheter method and order. Each bar represents mean ± SEM of data from 12 rats.

LPP measurements were not significantly different between both experimental groups (Fig. 3) indicating that there were no significant differences in LPP, baseline, or peak bladder pressure between suprapubic or transurethral catheterization. Removal or insertion of a transurethral catheter did not significantly change baseline, peak pressure or LPP (Fig. 3). Baseline pressure, peak pressure and LPP did not change significantly after initial catheter insertion (Fig. 3). There was also no significant interaction between order of catheter insertion and catheter method for peak pressure (Fig. 3). Although not significantly different, peak bladder pressure during LPP testing was visually higher in rats that underwent transurethral catheterization first (55.2 ± 5.1 cmH_2_O) and afterward underwent suprapubic catheterization (52.4 ± 5.4 cmH_2_O), compared to rats that underwent LPP testing using a suprapubic catheter first (41.2 ± 4.1 cmH_2_O) and a transurethral catheter second (45.0 ± 3.8 cmH_2_O). Likewise, LPP was increased when measured first with a transurethral catheter (44.1 ± 4.8 cmH_2_O) and afterward with a suprapubic catheter (40.1 ± 4.4 cmH_2_O) compared to measurement with a suprapubic catheter first (30.8 ± 4.8 cmH_2_O) and afterward with a transurethral catheter (33.8 ± 4.7 cmH_2_O), although these differences were also not statistically significant.

### External urethral sphincter electromyography (EUS EMG)

**Figure 4:**
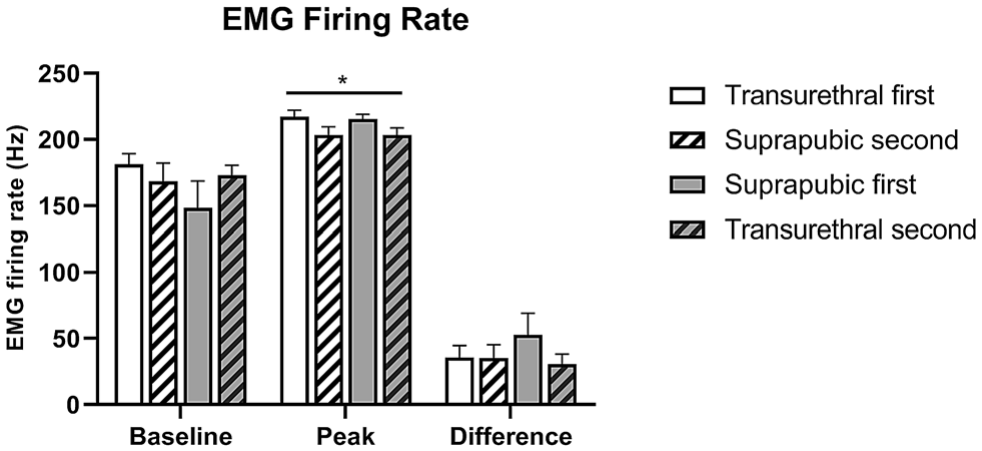
Firing rate of external urethral sphincter electromyography (EUS EMG) during leak point pressure (LPP) testing. EUS EMG firing rate at baseline and peak bladder pressure of LPP testing and the difference between the two grouped by order of catheterization. *Indicates that repeated measures ANOVA revealed a statistically significant effect of order on peak measurements (*p* < 0.05), indicating significant higher firing rate peak after initial catheter insertion, compared to subsequent catheter insertion. Each bar represents mean ± SEM of data from 12 rats.

**Figure 5:**
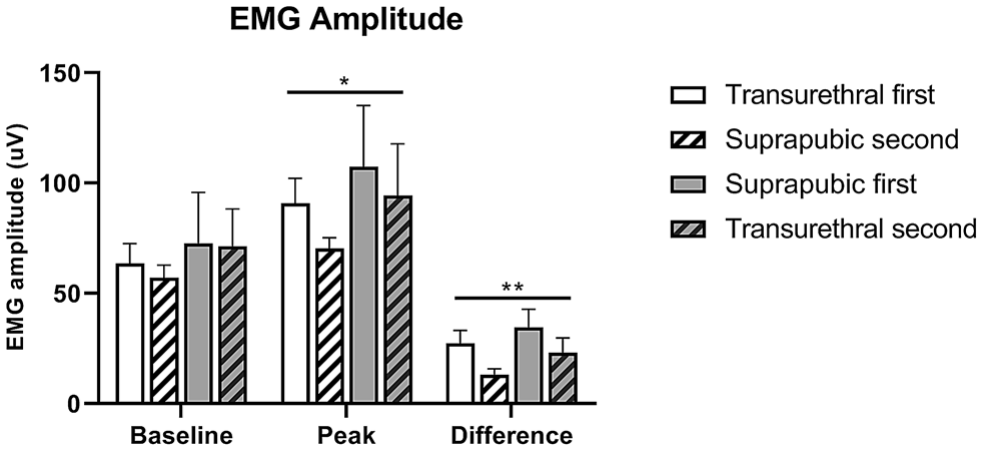
Amplitude of external urethral sphincter electromyography (EUS EMG) amplitude during leak point pressure (LPP) testing. EUS EMG amplitude at baseline and peak bladder pressure of LPP testing and the difference between the two grouped by order of catheterization. * and ** Indicate that repeated measures ANOVA revealed a statistically significant effect of order on peak and difference between peak and baseline measurements (*p < 0.05, **p < 0.01), indicating significantly higher peak amplitude and difference in amplitude between peak and baseline after initial catheter insertion, compared to subsequent catheter insertion. Each bar represents mean ± SEM of data from 12 rats.

In each experimental group, EUS EMG firing rate and amplitude increased from baseline to peak bladder pressure during LPP testing (Figs. 4, 5). There were no significant differences in EUS EMG results between measurements with a suprapubic catheter in situ versus a transurethral catheter (Figs. 4, 5), so catheter method did not affect EMG outcomes. However, EUS EMG peak firing rate, EUS EMG peak amplitude, and difference in amplitude between baseline and peak were significantly higher after the first compared to the second catheterizations (Figs. 4, 5), suggesting possible degradation of EUS function with extended testing duration.

EUS EMG firing rate at peak bladder pressure during LLP testing with initial catheterization was significantly higher (217.3 ± 5.0 Hz for transurethral first and 215.8 ± 3.3 Hz for suprapubic first, Fig. 4) than measurements taken with the subsequent catheterization (203.6 ± 6.2 Hz for suprapubic second and 203.5 ± 5.4 Hz for transurethral second, Fig. 4).

EUS EMG peak amplitude during LPP testing also decreased significantly during measurements with the second method (70.3 ± 4.9 µV for suprapubic catheterization second and 94.5 ± 23.3 µV for transurethral catheterization second, Fig. 5), compared to the initial method (90.8 ± 11.4 µV for transurethral catheterization first and 107.3 ± 27.0 µV for suprapubic catheterization first, Fig. 5). EUS EMG amplitude difference between baseline and peak likewise decreased significantly during measurements with the second catheter method (13.2 ± 2.6 µV for suprapubic catheterization second and 23.1 ± 6.7 µV for transurethral catheterization second, Fig. 5), compared to the initial method (27.3 ± 5.9 µV for transurethral catheterization first and 34.6 ± 28.2 µV for suprapubic catheterization first, Fig. 5).

## Discussion

In our study, we did not find that catheterization method affected urethral functional measurements. However, there were significant differences between first and second catheter insertions with significantly higher EUS EMG peak firing rate as well as peak EMG amplitude, and the difference between peak and baseline EMG amplitude in rats after initial catheterization compared to subsequent catheterization. These findings could indicate loss of EUS function with increasing measurement duration due to disruption of the integrity of the pelvic structures after cutting the pubic symphysis, which results in reduction of blood supply and denervation^13^. Alternatively, it could be an effect of continuing anesthesia or extended exposure of the urethra with time. Pressure drift, which can occur during cystometry^14^, did not affect our outcome data over time, since baseline bladder pressures during the first and second catheterizations did not differ.

Differences in EUS EMG amplitude at peak and difference between peak and baseline were more pronounced in rats having a suprapubic catheter initially. An explanation for this may be that insertion of a transurethral catheter caused urethral irritation, which resulted in an inflamed urothelium and EUS EMG deterioration.

Initial catheters were placed prior to exposing the urethra; therefore, initial transurethral catheter insertion was performed without visualization of the course of the urethra. In contrast, the second transurethral catheter insertion was performed after the urethra was exposed. This may have led to less traumatic insertion in the second group, inducing less irritation and edema of the urethral mucosal seal and decreased EUS activation, leading to significantly decreased EUS EMG amplitude with initial transurethral catheterization.

Our goal with this crossover designed study was to test both catheter methods within the same animal to be able to evaluate the direct effects of catheter method on LPP and EUS EMG. In most cystometry or LPP studies, only one catheterization method is chosen in the experimental design. However, both catheter methods can be used at the same time to measure bladder and urethral pressure or to improve testing accuracy by using one catheter for fluid infusion and one to measure bladder pressure.

In contrast to the results of our study, clinical studies have demonstrated that the presence of a transurethral catheter may affect measurement of urethral function^11,15,16^. Abdominal LPP, as measured with a rectal catheter or intravaginal catheter, significantly decreases during Valsalva and coughing after removal of a transurethral catheter in women, suggesting that the presence of a transurethral catheter may mask SUI^15^. Supporting this, 52% of women with symptoms of SUI who did not have urine leakage during urodynamic testing with a transurethral catheter in situ had leakage after removal of the catheter^11^. Catheter caliber has been demonstrated to influence Valsalva LPP since measurements with 8Fr catheters resulted in significantly greater LPP than measurements using 3Fr catheters^17^. The effects of urethral catheter presence was also studied in uroflow studies^18,19^. In female patients suffering from SUI, flow rate was reduced when using a transurethral catheter, compared to free uroflowmetry^18^. It has been suggested that this could be due to incomplete sphincter relaxation during voiding with the presence of a transurethral catheter^19^, which may relate to the significantly reduced EUS EMG amplitude if a transurethral catheter was used initially.

In contrast to animal studies, the diagnosis of SUI in women is based on patient history, questionnaires or a bladder diary in combination with objective urine loss during physical examination (positive stress test) or urodynamic testing^20^. Cough and Valsalva are used to increase abdominal pressure during urodynamic testing to induce leakage in the urodynamic laboratory setting^21^. However, urodynamic testing is not required for simple cases, because the predictive value is unclear and does not influence outcomes of surgery for SUI^22^. In contrast, for preclinical testing of novel therapies for SUI in animal models, LPP testing is crucial as it is the primary clinically relevant efficacy endpoint.

In prior studies in control rats, mean LPP using transurethral catheterization was 35–50 cmH_2_O^10,12,23^. In a study that tested LPP with a suprapubic catheter in normal rats, mean LPP was within that range at 44.3 cmH_2_O^24^. Likewise, LPP was approximately 40 cmH_2_O in animals 3 weeks after a sham injury in two different studies using transurethral^25^ or suprapubic catheters^26^, again within the range above. However, both catheter methods were not compared head-to-head in these studies. The data are therefore not completely comparable due to heterogeneous experimental methods.

Effects of catheter methods on cystometric parameters have been tested previously^27,28^. In prior studies, transurethral catheters resulted in higher maximum voiding pressure and lower flow rate, suggesting that there is a functional partial outlet obstruction after placement of a transurethral catheter^27^. Other studies measured cystometric outcomes using a urethral catheter, followed by a cystotomy catheter and showed significantly higher bladder pressure with transurethral catheters^28^. In these previous studies, in which transurethral and suprapubic catheter methods were tested in separate animals, results suggested direct urethral obstruction and alterations of outlet afferent sensitivity caused by insertion of a urethral catheter^29^. The cross-over design of our study may explain the difference between our findings and previous studies, since we were able to compare outcomes and order of catheterization within the same animals.

In this study, we only tested control animals. Rats with SUI could be tested in the future to clarify these results in the animal model. However, one might presume that transurethral catheters would be more likely to create a partial obstruction in a situation of greater urethral competency such as in control rats; thus normal rats could potentially suffice to demonstrate the difference between catheterization methods. Nonetheless, testing rats with SUI would be an important follow-up study to confirm our results in the animal model.

One of the limitations of our study is that we did not test the effects of catheter caliber to determine if a smaller caliber catheter could reduce the trauma of urethral insertion. In addition, our results are not generalizable to male rats, since transurethral catheter insertion is not possible in male rats without damaging the urethral integrity, since the penile urethra ends in a flexure^29^.

The use of anesthesia in rats may have affected bladder function compared to awake cystometry^30^; however, anesthesia is needed in animal studies for LPP and EUS EMG recordings. Anesthetics have been shown to impact urodynamic parameters in preclinical studies, by increasing bladder capacity, causing non-voiding contractions and decreasing changes in bladder pressure^31^. Urethane is generally preferred as it better maintains reflexes than other anesthetic agents^32,33^, including propofol, which suppresses bladder function to a greater extent than urethane anesthesia^34^.

We performed LPP measurements 6–12 times in each rat, at half of the bladder capacity measured in the first cystometry cycle. We have previously shown that LPP is not dependent on the volume in the bladder^32^. We used a filing rate of 5 mL/h, a supraphysiological filling rate that is commonly used in rat cystometry studies and can increase bladder pressure and decrease bladder capacity over time^35,36^. If supraphysiological filling rates had affected our measurements over time, there would have been a trend of lower LPP measurement, due to increased baseline bladder pressure, in the last measurements compared to the first. However, we did not observe this to occur in our data. A limitation of this study is that we did not record cystometry data; therefore we cannot show whether bladder function was affect by catheterization method.

Finally, we used quadruped animals and dissected the pelvis to be able to record EUS EMG, restricting comparison to clinical studies and investigations such as on timing of activation of pelvic floor muscles relative to LPP testing and EUS EMG activation. Nonetheless, this study is the first to compare two frequently used catheterization methods for measuring urethral function in rats and suggests a method for design and analysis to compare effects of catheterization on cystometry in rats in a future study. We demonstrated that urethral function as measured with LPP and simultaneous EUS EMG is not different whether using a suprapubic or transurethral catheter.

In conclusion, urethral function measurements are not affected by method of catheter insertion. However, two catheterization methods should not be combined within the same procedure since this will introduce order dependence and reduce testing accuracy. Either suprapubic or transurethral catheterization methods can be used for LPP testing, taking into account that EUS EMG measurements with a second method of catheterization can degrade with extensive duration of the study.

## Methods

### Study design

The Institutional Animal Care and Use Committee of the Louis Stokes Cleveland VA Medical Center approved this study. The study was carried out in compliance with the ARRIVE guidelines. All methods were carried out in accordance with relevant guidelines and regulations. Twelve virgin female Sprague-Dawley rats (Harlan Laboratories, Indianapolis, IN), aged 31–32 weeks and weighing 260–310 g, were included. Rats were housed with 12-h interchanging light–dark cycle and ad libitum access to food and water. Animals were randomly divided into two groups for initial catheterization to ensure that there were no significant differences in weight, age, sex, or parity between the groups. Urethral function, as measured by LPP and EUS EMG, was tested using transurethral and suprapubic catheters in a cross-over design (Fig. 1).

### Catheter implantation

Rats were anesthetized with urethane (1.2 g/kg) intraperitoneally, received 1% isoflurane anesthesia, and were placed supine. For suprapubic catheter insertion, the bladder dome was exposed via a midline incision between the most caudal mammary glands. A polyethylene catheter (PE-50) with a flared tip was inserted into the bladder and secured with a purse-string suture. The catheter was tunneled through the abdominal skin and connected to a syringe pump (Model 200; KD scientific, New Hope, PA) and pressure transducer (Model PT300; Astro-Med, Inc., Providence, RI). Air pressure at level of the bladder was used as reference for bladder pressure. A lubricated (Surgilube^®^, Fougera Pharmaceuticals Inc.) PE-50 catheter was inserted via the urethral meatus and connected to the syringe pump and pressure transducer for urodynamic measurements with a transurethral catheter. Saline was infused in the bladder at a rate of 5 mL/h.

### LPP testing and concomitant EUS EMG measurement

LPP testing with simultaneous EUS EMG measurements were conducted as described previously^32^. A horizontal incision directly cranial to the clitoral glands was made and both inferior epigastrial arteries and veins were ligated. The pubic bone was separated from the rectus abdominal muscles and the pubic symphysis was cut to expose the urethra underneath.

Bipolar parallel platinum electrodes (250 µm diameter, 1.0 mm apart) were positioned on the EUS and connected to an amplifier with band pass frequencies of 3 Hz–3 kHz (Model P511; AC Amplifier, Astro-Med, Inc., Providence, RI) and an electrophysiological recording system with 10 kHz sampling rate (PowerLab 8/35, ADInstruments).

Isoflurane anesthesia was discontinued, since it affects urethral function, due to suppression of EUS activity^37^. At half bladder capacity, bladder pressure and EUS EMG were recorded 3–6 times while pressing slowly on the abdomen, directly over the bladder to increase bladder pressure in the absence of a bladder contraction. Pressure was quickly removed after leakage from the urethral meatus was observed. Subsequently initial suprapubic catheters were disconnected, shortened and occluded and a transurethral catheter was placed. Likewise, initial transurethral catheters were removed and replaced with a suprapubic catheter. This was followed by a second series of LPP testing with EUS EMG recordings. After testing, rats were euthanized with a pentobarbital sodium and phenytoin sodium overdose (780 mg/kg and 100 mg/kg respectively) via intraperitoneal injection.

### Data analysis

Data with clear recording errors, such as those with electrode detachment during LPP measurement, were excluded from analysis. Peak pressure was defined as bladder pressure at leakage. Baseline pressure was the pressure immediately before applying pressure on the bladder. LPP was calculated by subtracting baseline from peak pressure. One-second segments of EUS EMG at baseline and peak pressure were selected for data analysis (LabChart7, ADInstruments). EMG signals had a threshold of 15 µV to reject noise and a notch filter at 60 and 120 Hz to remove electrical interference. EUS EMG firing rate is a measure of muscle fiber contraction or recruitment and amplitude is a measure of the muscle action potential^38^. Mean firing rate and amplitude were calculated as done previously^39^. Firing rate and amplitude differences between baseline and peak pressure were determined for each segment. Mean values for each animal were used for further statistical analysis.

### Statistical analysis

The experimental design used for this study is a full two level, three variable study run in triplicate across 12 animals for a total of 24 experiments. The variables of interest were catheter type, order of catheter application to a specific animal and observer. The design permitted an assessment of catheter type, catheter order, the interaction of type and order differences, and observer differences. Because each animal was tested using both catheters, the final measurements are repeated measures and the method used for the analysis was repeated measures ANOVA. Initial analysis indicated that the effect of observer differences was non-significant. As a result, the factor for observers was dropped from the model. This allowed the degrees of freedom associated with observer to be added to the error term, improving precision of its estimate.

An assessment of the data indicated all of the measured responses were acceptably normal and, with the exception of EMG firing rate, all were homoscedastic across catheter type. The heteroscedasticity for EMG firing rate was driven by a single outlier data point which was removed and not used in the analysis of EMG firing data. Statistical significance was set at *p* < 0.05. Data are presented as mean ± standard error of the mean (SEM). All analysis was done using SAS 9.4. Graphs were drafted in GraphPad Prism 6, GraphPad Software Inc.

## Data Availability

The dataset generated and analyzed during this study is available from the corresponding author on reasonable request.
